# Effective Mn-Doping in AgInS_2_/ZnS Core/Shell Nanocrystals for Dual Photoluminescent Peaks

**DOI:** 10.3390/nano9020263

**Published:** 2019-02-14

**Authors:** Ryo Sakai, Hikaru Onishi, Satomi Ido, Seiichi Furumi

**Affiliations:** Department of Applied Chemistry, Faculty of Science, Tokyo University of Science (TUS), 1-3 Kagurazaka, Shinjuku, Tokyo 162–8601, Japan; 1317654@ed.tus.ac.jp (R.S.); b116635@alumni.tus.ac.jp (H.O.); b116623@alumni.tus.ac.jp (S.I.)

**Keywords:** semiconductor, nanocrystals, silver indium sulfide, photoluminescence, manganese, dopant

## Abstract

We developed the effective Mn-doping procedure for AgInS_2_(AIS)/ZnS core/shell nanocrystals (NCs) to exhibit dual photoluminescence (PL) peaks. Although the AIS/ZnS core/shell NCs showed solely a single PL peak at ~530 nm, incorporation of a small amount of Mn as a dopant within the AIS/ZnS NCs resulted in the simultaneous emergence of dual PL peaks at ~500 nm (green PL) arising from AIS/ZnS NCs and ~600 nm (orange PL) from the Mn dopants. Furthermore, we succeeded in significantly increasing the absolute PL quantum yield value of dual emissive AIS/ZnS NCs incorporated with Mn dopants from 10% to 34% after surface passivation with another ZnS shell for the formation of core/shell/shell structures.

## 1. Introduction

Colloidal semiconductor nanocrystals (NCs) have, over the past two decades, been attracting considerable interest as intriguing nanomaterials from both fundamental and technological viewpoints of the nanotechnology [1,2,3]. One of the majorly appealing features of semiconductor NCs is that they exhibit intrinsic and unique optoelectronic characteristics at the nanometer size. For instance, photoluminescence (PL) peak is tunable by the nanometer-size of semiconductor NCs owing to the quantum size effect. The significant progress in semiconductor NCs has established a wide variety of methodologies for the syntheses of high-quality NCs from metal precursors in the liquid phase. Chalcogenide-type binary semiconductor NCs such as CdS, CdSe and PbS, which exhibit typically the quantum size effect, are the most frequently investigated examples of NCs [4,5,6]. When the outermost surface of semiconductor NCs is generally overcoated by wide-bandgap inorganic semiconductors such as ZnS and ZnSe to yield the core/shell structures, higher values of absolute PL quantum yield (PL-QY) can be realized by the passivation of non-radiative recombination sites on their surface [7,8]. Furthermore, much attention has been paid to the technological applications such as light-emitting diodes [9,10,11,12], photovoltaic devices [13,14,15,16,17] and biological imaging makers [18,19]. However, these binary semiconductor NCs cannot be implement into practical applications in a straightforward way. At present, these toxic elements are regulated by the Restriction of Hazardous Substances (RoHS) directive. Therefore, more emphasis in this research field is placed on the development of alternative luminescent nanomaterials that can exclude the highly toxic elements.

In this context, the recent progress in the field of semiconductor NCs has established the syntheses and optoelectronic applications of chalcopyrite-type ternary semiconductor NCs such as AgInS_2_ (AIS), CuInS_2_ (CIS) and so forth due to their relatively low-toxic and environmentally-friend nanomaterials [20,21,22,23,24,25,26,27]. Previously, we have successfully synthesized luminescent AIS NCs through pyrolysis of low-toxic Ag and In acetate precursors [28]. Such AIS NCs exhibited a high absolute PL-QY value of ~60% and their permanent stability for over 21 months. However, the AIS NCs showed solely a single PL peak around 600 nm as orange PL. Considering the practical applications to white light-emitting diodes, it is important to prepare luminescent NCs with a wide PL wavelength range. This motivated us to synthesize AIS NCs with dual PL peaks, for instance, green and orange PL by incorporating optically active dopants such as transition metal ions into the NCs.

In this paper, we report the effective procedure to incorporate a small amount of Mn dopant into AIS/ZnS core/shell NCs, which leads to dual PL peaks at ~500 nm (green PL) and ~600 nm (orange PL). After the AIS core NCs are reacted with Zn and S precursors, the single PL peak is blue-shifted from ~600 nm to ~530 nm owing to the shell formation of larger bandgap ZnS covered on the AIS NCs. Subsequently, by incorporating Mn dopants into the AIS/ZnS NCs, the PL spectral shape of the Mn-doped AIS/ZnS (AIS/ZnS:Mn) NCs is remarkably altered to exhibit a broad PL band with dual peaks at ~500 nm and ~600 nm. At this stage, the absolute PL-QY has quite a low value of 10%. However, we succeed in distinct enhancement of the absolute PL-QY values from 10% to 34% by passivating another ZnS shell layer on the AIS/ZnS:Mn NC surface for the formation of AIS/ZnS:Mn/ZnS NCs as core/shell/shell structures.

## 2. Experimental Section

### 2.1. Synthesis of AIS Core NCs

The AIS core NCs were synthesized by the pyrolysis of low-toxic Ag and In precursors according to our previous report [28]. Silver(I) acetate (0.1 mmol) and indium(III) acetate (0.6 mmol) were dissolved in 1-octadecene (ODE; 24 mL) in the presence of oleic acid (OA; 1.2 mmol), tri-*n*-octylphosphine (TOP; 1.3 mmol) and 1-dodecanethiol (DDT; 30 mmol) as the organic ligands. This mixture was heated from 120 °C to 190 °C to facilitate the pyrolysis of Ag and In precursors. After gentle cooling, the solution was washed with ethanol several times to remove excess OA, TOP, DDT, and other byproducts. Subsequently, the precipitate was dried and dispersed in spectrophotometrically graded toluene. Finally, the toluene solution was filtered through polytetrafluoroethylene (PTFE) membrane filters with 0.2 µm pore size.

### 2.2. Syntheses of AIS/ZnS Core/Shell NCs and AIS/ZnS:Mn Core/Shell NCs

The powdered AIS core NCs, synthesized in the preceding section, were dissolved in ODE (13 mL) with DDT (8.3 mmol) as the ligand by heating to 120 °C. Subsequently, the solution in a PTFE vessel was mixed by adding an ODE (7.3 mL) solution containing zinc(II) stearate (ZnSt_2_; 0.4 mmol) and oleylamine (9.1 mmol) in a glovebox filled with Ar for an inert atmosphere. After the PTFE vessel was set in an autoclave, we elevated the temperature to 230 °C and maintained it for 3 h. After cooling, the resultant solution was also washed with ethanol, dispersed in toluene, and filtrated with PTFE filters.

For the preparation of AIS/ZnS:Mn core/shell NCs, the powdered AIS core NCs were also reacted by adding not only ZnSt_2_ (0.4 mmol), but also manganese(II) stearate (0.08 mmol) as the Mn precursor in an autoclave [29,30], according to the same procedure of AIS/ZnS core/shell NCs mentioned above in this section.

### 2.3. Synthesis of AIS/ZnS:Mn/ZnS Core/Shell/Shell NCs

The AIS/ZnS:Mn core/shell NCs were also reacted by adding a solution of ZnSt_2_ (0.8 mmol) and OA (0.3 mmol) in ODE (7.3 mL). The reaction mixture in a PTFE vessel was heated to 230 °C for 2 h in an autoclave in order to form the AIS/ZnS:Mn/ZnS core/shell/shell NCs. Subsequently, the resultant solution was also washed with ethanol, dispersed in toluene and filtrated with PTFE filters.

### 2.4. Characterizations

X-ray diffraction (XRD) patterns were measured by an X-ray diffractometer (Rigaku, Tokyo, Japan, RINT-2500) using Cu Kα radiation under the operating condition of 50 kV and 300 mA for analyses of crystal structures of AIS-based NCs. The size and shape of as-synthesized NCs were observed by a transmission electron microscope (TEM; JEOL, Tokyo, Japan, JEM-2100 and JEM-2100F) at 200 kV. Microscopic elemental mapping images of Zn and Mn atoms in AIS/ZnS:Mn NCs and AIS/ZnS:Mn/ZnS NCs were obtained by an atomic resolution electron microscope (ARM; JEOL, Tokyo, Japan, JEM-ARM200F), a kind of TEM, at 200 kV. By using an inductively coupled plasma-mass spectrometer (ICP-MS) (Agilent Technologies, Santa Clara, CA, USA, Agilent 7500), we calculated atomic molar ratios of Zn and Mn.

UV-visible absorption spectra of as-synthesized NCs solutions were obtained on a photodiode-arrayed absorption spectrophotometer (Agilent Technologies, Santa Clara, CA, USA, Agilent 8453). Steady-state PL spectra were measured with a fluorescence spectrophotometer (Shimadzu Corporation, Kyoto, Japan, RF-5300 PC). Time-resolved PL lifetimes and decay profiles were obtained by a fluorescence lifetime spectrometer (Hamamatsu Photonics, Shizuoka, Japan, Quantaurus-Tau C11367-01) based on the time-correlated single-photon counting technique. The excitation wavelength was set at 365 nm. The experimental PL decay profiles were deconvoluted and fitted by setting appropriate parameters. Absolute PL-QY values were recorded on an absolute PL quantum yield measurement system (Hamamatsu Photonics, Shizuoka, Japan, C9920-02G) equipped with a multichannel spectrometer, Xe lamp and integrating sphere. Briefly, the absolute PL-QY value is defined as *PN*_PL_/*PN*_Abs_, where *PN*_PL_ means the photon number of PL from our AIS-based NCs and *PN*_Abs_ is the photon number absorbed by the NCs. This measurement system was beforehand calibrated by using an ethanolic solution of a standard anthracene purified by the zone melting procedure. We confirmed that this standard anthracene solution shows an absolute PL-QY value of 27 ± 3% by optical excitation with 355 nm light [31,32]. For precise evaluation of the absolute PL-QY values, we adjusted the absorbances of solutions of all NCs at 365 nm near 0.1. All measurements were carried out at room temperature.

## 3. Results and Discussion

**Figure 1:**
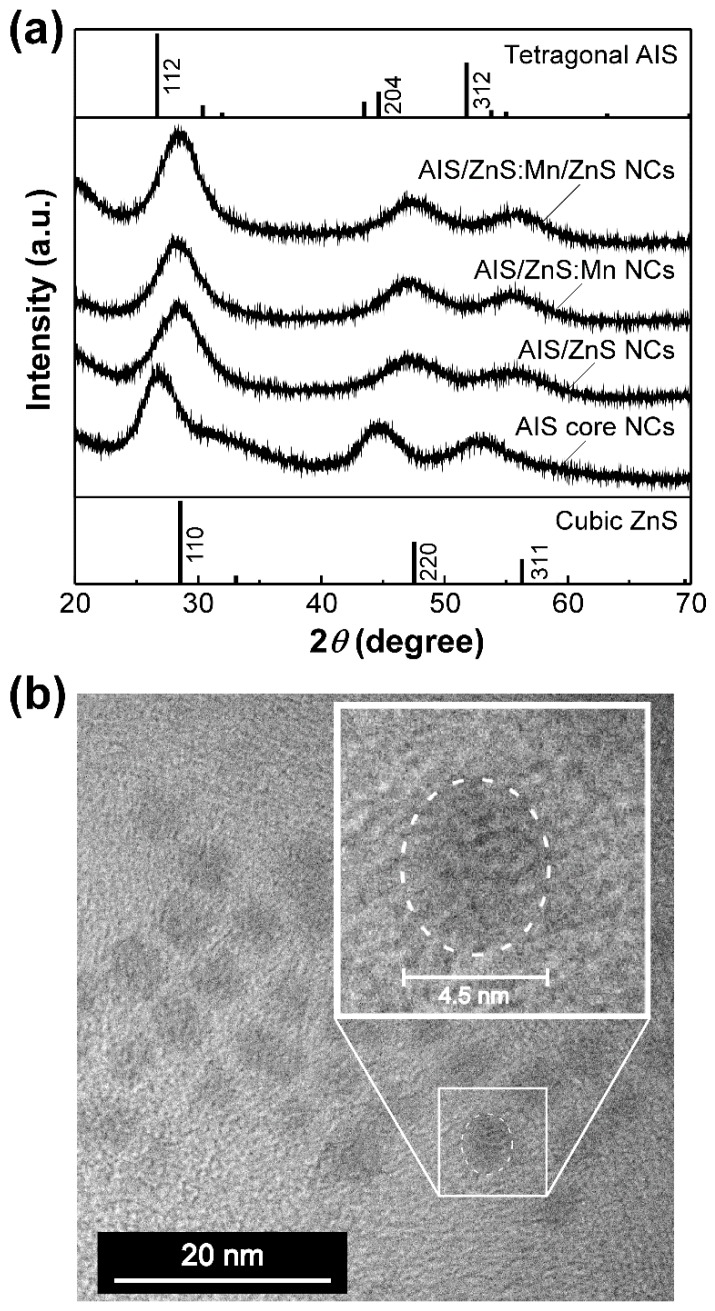
(**a**) XRD patterns of AgInS_2_(AIS) core NCs, AIS/ZnS core/shell NCs, AIS/ZnS:Mn core/shell NCs and AIS/ZnS:Mn/ZnS core/shell/shell NCs. For comparison, upper and lower profiles in this figure are the XRD patterns for bulk materials of tetragonal AgInS_2_ (PDF# 00­0251330), cubic ZnS (PDF# 00­0050566), respectively. (**b**) TEM image of AIS/ZnS:Mn/ZnS NCs. The inset denotes the magnified image of circularly dashed area.

First, we measured the XRD patterns of our AIS-based NCs to elucidate the structural characterization. Figure 1a shows the XRD patterns of AIS core NCs, AIS/ZnS core/shell NCs, AIS/ZnS:Mn core/shell NCs and AIS/ZnS:Mn/ZnS core/shell/shell NCs. All the XRD patterns exhibited broad XRD peaks arising from relatively small size of the NCs in the range of 2–4 nm, as will be described below. The AIS core NCs showed three XRD peaks at 2*θ* of ~27°, ~43° and ~52°, adequately corresponding to the diffractions of (112), (204) and (312) planes of bulked tetragonal AgInS_2_ crystal, respectively. For the AIS/ZnS core/shell NCs, the three XRD peaks shifted to larger angles of 2*θ*. The shifted peaks located near the diffractions of (111), (220) and (311) planes of bulked cubic ZnS crystal, indicating that the AIS core NCs are covered with a relatively thick ZnS shell layer. The AIS/ZnS:Mn NCs exhibited a similar XRD pattern as that of AIS/ZnS NCs. Therefore, it is plausible that the crystalline lattice of ZnS shell of AIS/ZnS:Mn NCs can be preserved without deterioration even after the formation of ZnS shell containing Mn dopants. Such an experimental result was consistent with some precedents of other nanocrystals such as ZnS and Zn-alloyed CIS with Mn dopants [33,34,35]. Additionally, when the AIS/ZnS:Mn NCs were overcoated with a ZnS shell layer to from AIS/ZnS:Mn/ZnS core/shell/shell NCs, the three XRD peaks shifted closer to the diffractions of bulked cubic ZnS crystal rather than the AIS/ZnS and AIS/ZnS:Mn NCs. This result suggests that the shell of AIS/ZnS:Mn/ZnS NCs is thicker than those of AIS/ZnS and AIS/ZnS:Mn NCs.

TEM observations provide morphological information on the size and shape of NCs at the nanometer scale (Figure 1b and Figure S1, Supplementary Materials). Although the AIS core NCs showed the average size of 2.3 ± 0.2 nm, we found that the sizes of AIS/ZnS and AIS/ZnS:Mn NCs are 3.5 ± 0.4 nm and 3.4 ± 0.4 nm, respectively, by overcoating the ZnS shell layer on AIS NCs (Figure S2, Supplementary Materials). Figure 1b shows the TEM image of AIS/ZnS:Mn/ZnS NCs, confirming that the NCs adopt spherical shape with a size of 4.3 ± 0.5 nm. As compared with the AIS/ZnS:Mn NCs, the size of AIS/ZnS:Mn/ZnS NCs increased from ~3.4 nm to ~4.3 nm by overcoating with another ZnS shell on the AIS/ZnS:Mn NCs. Such an increase of NC size observed by TEM was compatible with the above-mentioned XRD results of AIS/ZnS:Mn/ZnS NCs.

**Figure 2:**
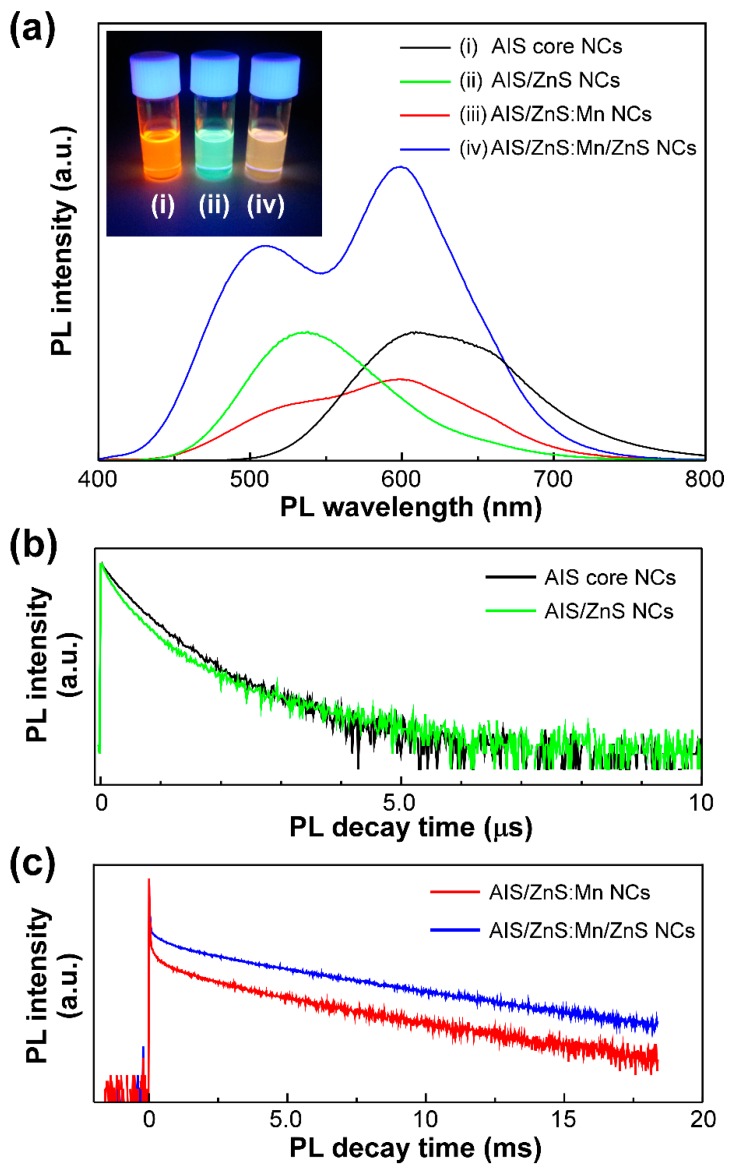
(**a**) PL spectra of solutions of AIS NCs (i: black), AIS/ZnS NCs (ii: green), AIS/ZnS:Mn NCs (iii: red), and AIS/ZnS:Mn/ZnS NCs (iv: blue) by optical excitation with 365 nm light. The inset represents the PL color image of solutions of AIS NCs, AIS/ZnS NCs, and AIS/ZnS:Mn/ZnS NCs. (**b**) PL decay curves of solutions of AIS NCs and AIS/ZnS NCs by optical excitation with 365 nm light. The PL decay measurements of AIS NCs and AIS/ZnS NCs were detected at 608 nm and 538 nm, respectively. (**c**) PL decay curves of solutions of AIS/ZnS:Mn NCs and AIS/ZnS:Mn/ZnS NCs by optical excitation with 365 nm light. The PL decay measurements of both AIS/ZnS:Mn NCs and AIS/ZnS:Mn/ZnS NCs were detected at 600 nm.

We then measured the PL spectra of solutions of AIS, AIS/ZnS, AIS/ZnS:Mn, and AIS/ZnS:Mn/ZnS NCs by optical excitation with 365 nm light. It turned out that our AIS-based NCs show the PL characteristics that are quite different from each other. As shown in Figure 2a, the AIS core NCs exhibited a broad PL band with only a single peak at ~600 nm. We could observe clear orange PL color (Inset of Figure 2a). According to our previous report [28], the PL peak of AIS core NCs is derived from the donor and accepter pair (DAP) recombination. The DAP recombination occurs when electron at donor recombines with hole at accepter and the recombination energy is given by

hν=Eg−(Ea+Ed)+e2εr

where $E_{g}$ is the bandgap energy, $E_{a}$ and $E_{d}$ are the donor and acceptor binding energies respectively, ε is dielectric constant, and r is the distance between electron and hole. As can be seen in the above formula, the DAP recombination energy depends on r. The DAP recombination energy shows relatively wide range, arising from a variety of donor-accepter distance, resulting in a broad PL band [36,37,38]. Furthermore, as measured absolute PL-QY of the AIS core NCs, the value reached ~64%, which was comparably high among those of the previously reported AIS NCs [36,37,38,39,40,41,42,43,44,45].

When the AIS core NCs were covered by a ZnS shell layer, we found a blue-shift of the single PL peak from ~600 nm (orange PL) to ~530 nm (green PL). We confirmed the PL color change from orange to green (Inset of Figure 2a). By measuring the absorption spectra, we also found a blue-shift of the absorption tail (Figure S3, Supplementary Materials). Such a blue-shift would result from that the energy gap increases by formation of the ZnS shell. This phenomenon can be interpreted by the hypothesis that the size of AIS core NCs reduces or the composited element ratio within NCs is changed by the surface reconstruction upon coating the AIS NCs with a ZnS shell [44,46,47]. With regard to the absolute PL-QY values, our AIS/ZnS core/shell NCs showed a much lower value of ~26% than that of the AIS core NCs. In our AIS/ZnS NCs, an excess amount of ZnS shell was formed on the AIS core NCs, as confirmed by XRD result (Figure 1a). At this time, many surface defects on the AIS core NCs in AIS/ZnS NCs emerged due to the excess amount of ZnS shell rather than those of as-prepared AIS core NCs covered with DDT molecules [48]. As compared with the previous reports, we used herein a large amount of DDT for the synthesis of AIS/ZnS NCs. Therefore, the DDT molecules hampered sufficient surface passivation of nonradiative sites on the AIS core NCs created during ZnS shelling, resulting in the decrease of absolute PL-QY value from ~64% to ~26%.

As the AIS core NCs were covered by a ZnS shell layer with Mn dopants, the PL spectral shape was remarkably altered to be a broad PL band with two peaks at both ~500 nm (green PL) and ~600 nm (orange PL). As will be discussed below, the former originated from PL of AIS/ZnS NCs, and the latter was from Mn emission by d-d transition. Moreover, this AIS/ZnS:Mn NCs showed quite low absolute PL-QY value of 10%. Therefore, we could not observe a clear PL color. This decrease of absolute PL-QY value can be explained by the hypothesis that surface defects of NCs by Mn-doping act as non-radiative recombination sites [46].

Subsequently, the AIS/ZnS:Mn NCs were overcoated with another ZnS shell layer for enhancement of the absolute PL-QY value. After forming the AIS/ZnS:Mn/ZnS NCs as core/shell/shell structures, the PL spectral shape was maintained as a broad PL band with two peaks at ~500 nm and ~600 nm. Of interest was the significant enhancement of absolute PL-QY values from 10% to 34%. The PL was found to be whitish orange in the color (Inset of Figure 2a). This is because the surface defects of AIS/ZnS:Mn NCs are covered with another ZnS shell, resulting in the formation of radiative recombination sites [46,49]. Even after prolonged storing for 14 months, we found clear whitish orange PL (Inset of Figure S4, Supplementary Materials). At this time, the PL spectral shape also showed two peaks at both ~500 nm and ~600 nm, and the absolute PL-QY value was 35% without deterioration of the quality (Figure S4, Supplementary Materials). As evident from the results, our AIS/ZnS:Mn/ZnS NCs exhibited excellent stability of PL properties for a long time.

To identify the PL components, we attempted to measure the time-resolved PL lifetimes of our AIS-based NCs. Generally, the semiconductor NCs show typically fast PL lifetimes with few hundreds of nanoseconds, whereas the Mn emission corresponds to the d-d transition (^4^T_1_ → ^6^A_1_) within the Mn d multiplets, leading to long lifetimes in the range of several hundreds of microseconds [50,51]. We measured the PL decay curves of AIS NCs and AIS/ZnS NCs at the PL wavelength of 608 nm and 538 nm, respectively, as shown in Figure 2b. By theoretically fitting the decay curves, the PL lifetimes of AIS NCs and AIS/ZnS NCs were estimated to be 390 ns and 340 ns, respectively, due to the DAP recombination [28,48,52]. Figure 2c shows the PL decay curves of AIS/ZnS:Mn NCs and AIS/ZnS:Mn/ZnS NCs measured at the PL wavelength of 600 nm, indicating relatively long PL lifetimes of 2.1 ms and 3.4 ms, respectively. As analyzed in more detail, there were fast and slow PL components in the decay curves. To elucidate two PL components, we measured the PL decay curves of AIS core NCs, AIS/ZnS NCs and AIS/ZnS:Mn/ZnS NCs at the PL wavelengths of 608 nm, 538 nm and 508 nm, respectively (Figure S5, Supplementary Materials). It turned out that the PL lifetime originating from AIS/ZnS in AIS/ZnS:Mn/ZnS NCs is 320 ns, which is adequately equal to that from AIS/ZnS NCs at 538 nm (Figure 2b). Therefore, such a fast PL component of AIS/ZnS NCs would be measured at longer PL wavelength of 600 nm (Figure 2c). As compared with two PL decay curves in Figure 2c, we found that the AIS/ZnS:Mn/ZnS NCs show a slightly longer PL lifetime of 3.4 ms rather than AIS/ZnS:Mn NCs. Such a prolonged PL lifetime emerges as a result of elimination of the surface defects on AIS/ZnS:Mn NCs by the formation of core/shell/shell structure. Therefore, the inert Mn ions near surface would be successfully overcoated by another ZnS shell layer to convert the radiative recombination sites.

**Figure 3:**
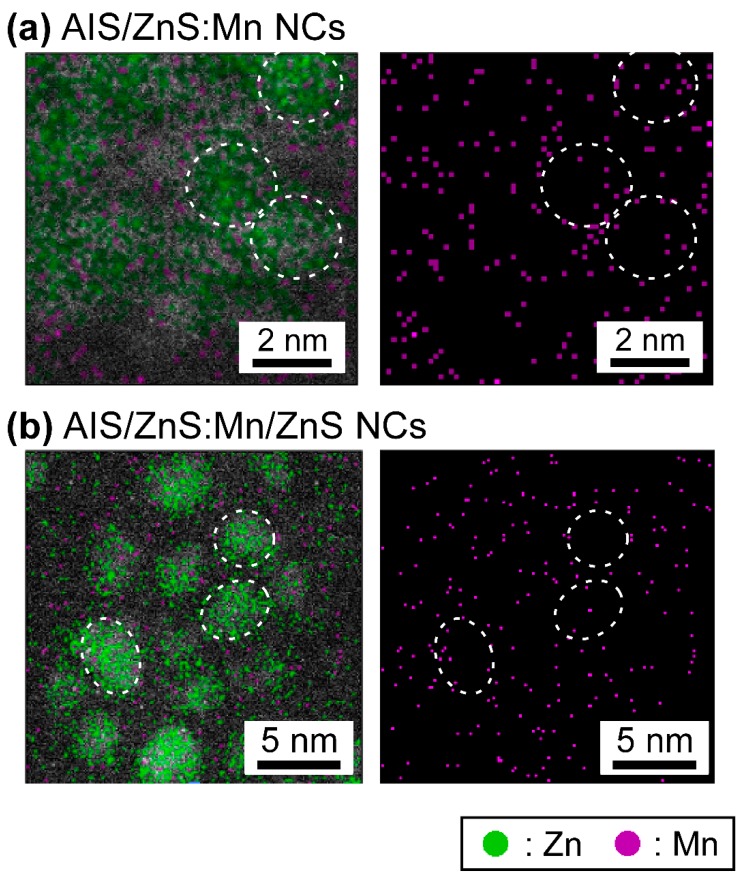
Microscopic elemental mapping images of AIS/ZnS:Mn NCs (**a**) and AIS/ZnS:Mn/ZnS NCs (**b**) observed by the ARM. Green and purple dots in the images indicate Zn and Mn, respectively, and the circular spots correspond to the single NCs.

Finally, the ARM observations enable to visualize the microscopic distribution of specific elements in the TEM images. Figure 3 shows the mapping images of Zn and Mn atoms within AIS/ZnS:Mn NCs and AIS/ZnS:Mn/ZnS NCs, wherein Zn and Mn mapping is indicated by green and purple dots, respectively. As given in Figure 3a, it was found that Zn atoms locate on single NCs, and almost Mn atoms existed on or near the outermost surface of AIS/ZnS:Mn NCs. In contrast, as shown in Figure 3b, we observed that the density of Mn atoms on or near surface decrease after overcoating with another ZnS shell layer on the AIS/ZnS:Mn NCs. This phenomenon was supported by the ICP-MS analysis results that the Mn/Zn atomic ratio of AIS/ZnS:Mn NCs critically decreases from 10% to 0.1% after the formation of AIS/ZnS:Mn/ZnS NCs. On account of the ARM and ICP-MS results, it is plausible that the Mn atoms on AIS/ZnS surface are exchanged by Zn atoms upon overcoating with another ZnS shell layer on the AIS/ZnS:Mn NCs. Consequently, the remaining Mn atoms are effectively covered by another ZnS shell to increase the radiative recombination sites, thereby intensifying the absolute PL-QY values from 10% to 34% by the formation of core/shell/shell structure.

Furthermore, in order to prove the AIS/ZnS:Mn/ZnS NCs as product, we analyzed the mapping images of Ag and In atoms in the same area of Figure 3b (Figure S6, Supplementary Materials) and the other areas (Figure S7, Supplementary Materials). Considering the results, we found that Ag and In atoms also exist within the NCs. The results provide clear evidence that our product correspond to AIS/ZnS:Mn/ZnS NCs, but not binary mixtures of ZnS:Mn and AIS/ZnS NCs.

## 4. Conclusions

We have successfully demonstrated the synthesis of Mn-doped AIS/ZnS NCs with simultaneous emergence of dual PL peaks at not only ~500 nm (green PL) arising from AIS/ZnS NCs, but also ~600 nm (orange PL) from Mn dopants. When the Mn-doped AIS/ZnS NCs were overcoated by another ZnS shell layer on the NCs for the formation of core/shell/shell structure such as AIS/ZnS:Mn/ZnS NCs, the absolute PL-QY value was intensified from 10% to 34%. The ARM observations provided us the interesting phenomena as follows. Another ZnS shell layer reduces the non-radiative recombination sites originating from Mn near the surface of NCs. Then, the remaining Mn atoms are effectively covered by ZnS shell to form the radiative recombination sites, eventually resulting in enhancement of the absolute PL-QY values.

The AIS/ZnS:Mn/ZnS NCs presented here are not optimal, however, and the optimization of synthetic procedures and structures of NCs would lead to technologically relevant PL performances. The present report opens promising ways to design and synthesize the dual emissive AIS-based NCs for the realization of next-generation white light-emitting diodes with high color rendering. Moreover, from biological viewpoint, long lifetime of excited-state of transition metals such as Mn and Cu leads to the potential application of efficient Förster resonance energy transfer from the NC donors to organic dye acceptors for the explanation of biological events using greener and safer NCs rather than binary semiconductor NCs such as CdS, CdSe and PbS [53].

## Supplementary Materials

The following are available online at http://www.mdpi.com/2079-4991/9/2/263/s1, Figure S1: TEM images of AIS core NCs, AIS/ZnS NCs and AIS/ZnS:Mn NCs, Figure S2: Histograms of size distributions of AIS core NCs, AIS/ZnS NCs, AIS/ZnS:Mn NCs and AIS/ZnS:Mn/ZnS NCs, Figure S3: Absorption spectra of solutions of AIS core NCs, AIS/ZnS NCs, AIS/ZnS:Mn NCs and AIS/ZnS:Mn/ZnS NCs in toluene, Figure S4: PL spectrum of a solution of AIS/ZnS:Mn/ZnS NCs in toluene after storing for 14 months since the synthesis, and the PL color image of the solution of AIS/ZnS:Mn/ZnS NCs, Figure S5: PL decay curves of solution of AIS core NCs, AIS/ZnS NCs, and AIS/ZnS:Mn/ZnS NCs by optical excitation with 365 nm light, Figure S6: Microscopic elemental mapping images of AIS/ZnS:Mn/ZnS NCs observed by the ARM, Figure S7: Microscopic elemental mapping images of AIS/ZnS:Mn/ZnS NCs observed by the ARM.
