# Supplementary material for: Effective Mn-Doping in AgInS2/ZnS Core/Shell Nanocrystals for Dual Photoluminescent Peaks
Source: Nanomaterials (Basel). 2019 Feb 14;9(2):263. doi: 10.3390/nano9020263 (PMC6410175; doi:10.3390/nano9020263)
Supplement: Supplementary file 1 [file nanomaterials-09-00263-s001.pdf]

Submitted to *Nanomaterials* as a Communication

Supplementary Materials

Effective Mn-Doping in $AgInS_2$/ZnS Core/Shell Nanocrystals for Dual Photoluminescent Peaks

Ryo Sakai, Hikaru Onishi, Satomi Ido, and Seiichi Furumi*

Department of Applied Chemistry, Faculty of Science,
Tokyo University of Science (TUS)
1–3 Kagurazaka, Shinjuku, Tokyo 162–8601, Japan
*Correspondence: furumi@rs.tus.ac.jp

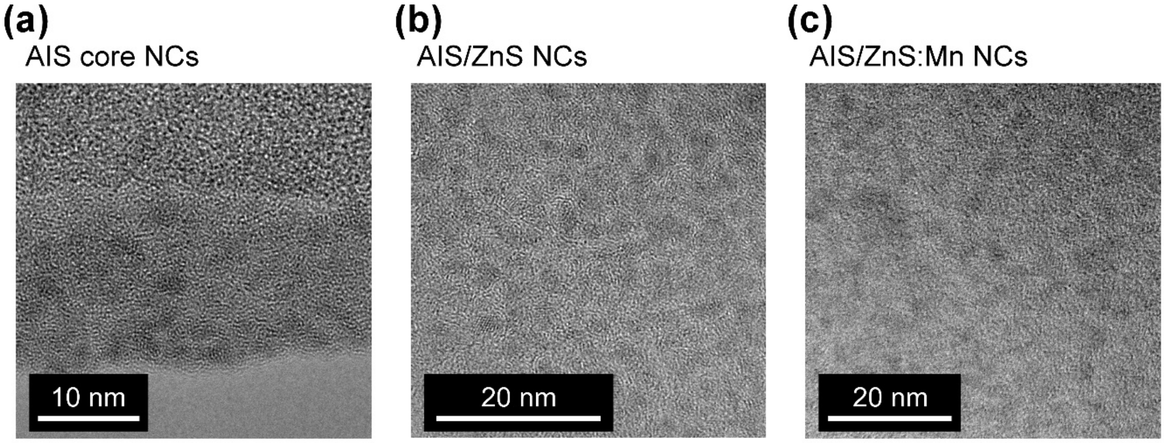

Figure S1. TEM images of AIS core NCs (a), AIS/ZnS NCs (b), and AIS/ZnS:Mn NCs (c).

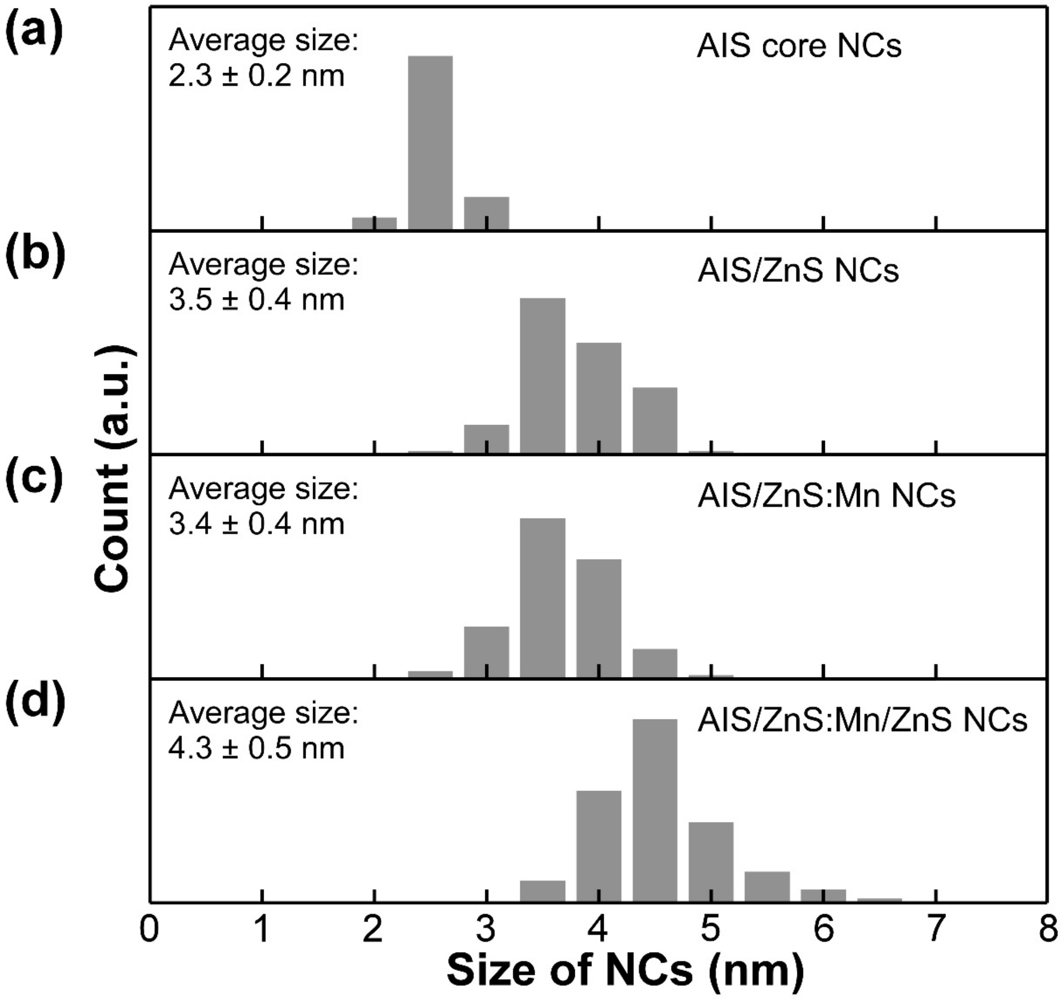

Figure S2. Histograms of size distributions of AIS core NCs (a), AIS/ZnS NCs (b), AIS/ZnS:Mn NCs (c), and AIS/ZnS:Mn/ZnS NCs (d). The NC sizes were estimated by counting over 100 NCs in the appropriate TEM images.

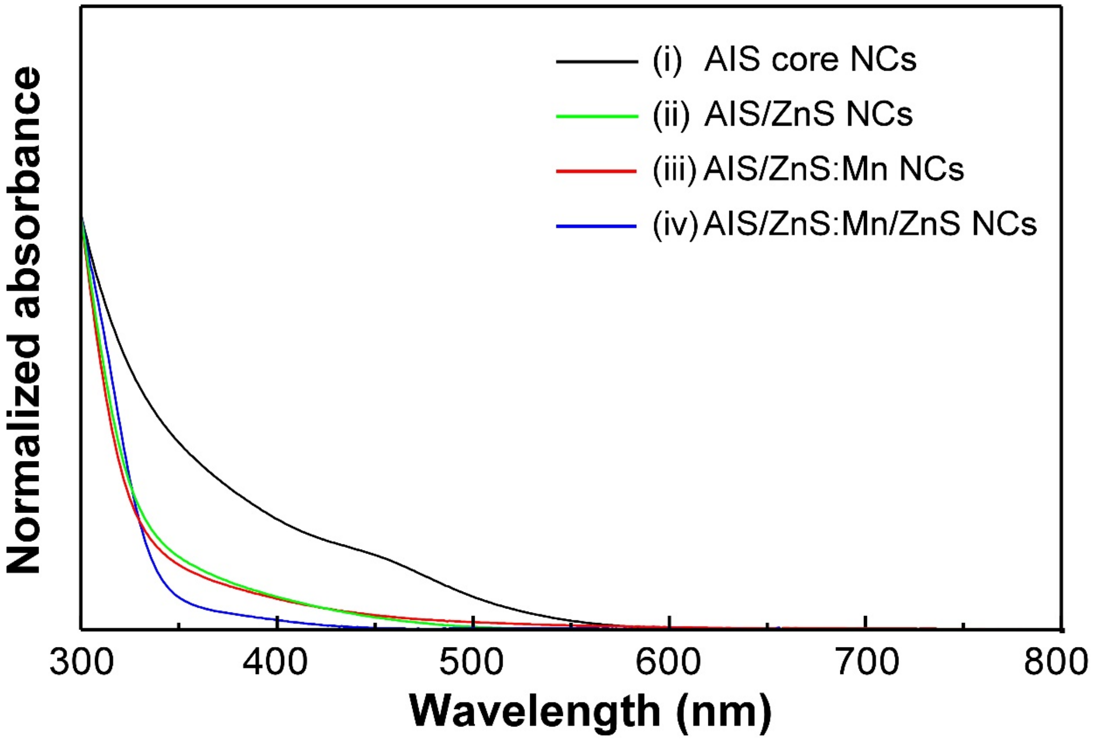

Figure S3. Absorption spectra of solutions of AIS core NCs (i), AIS/ZnS NCs (ii), AIS/ZnS:Mn NCs (iii), and AIS/ZnS:Mn/ZnS NCs (iv) in toluene.

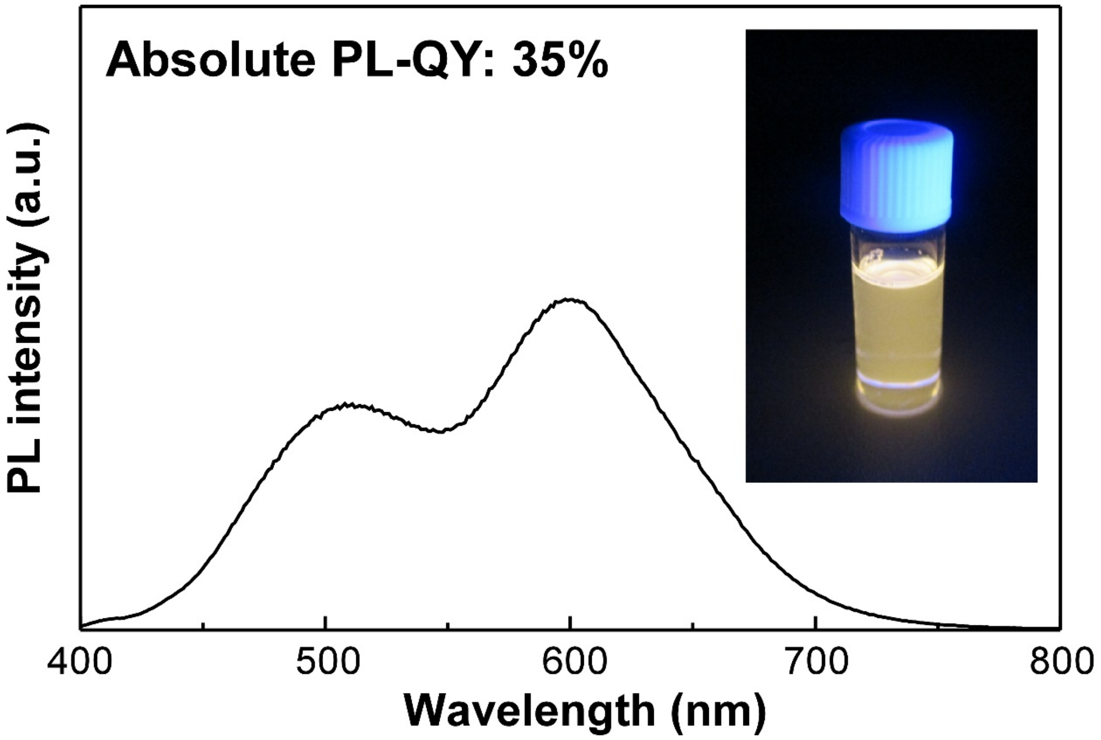

Figure S4. PL spectrum of a solution of AIS/ZnS:Mn/ZnS NCs in toluene after storing for 14 months since the synthesis. The excitation wavelength was also set at 365 nm. At this time, the absolute PL-QY value was estimated to be 35%. The inset shows the PL color image of the solution of AIS/ZnS:Mn/ZnS NCs.

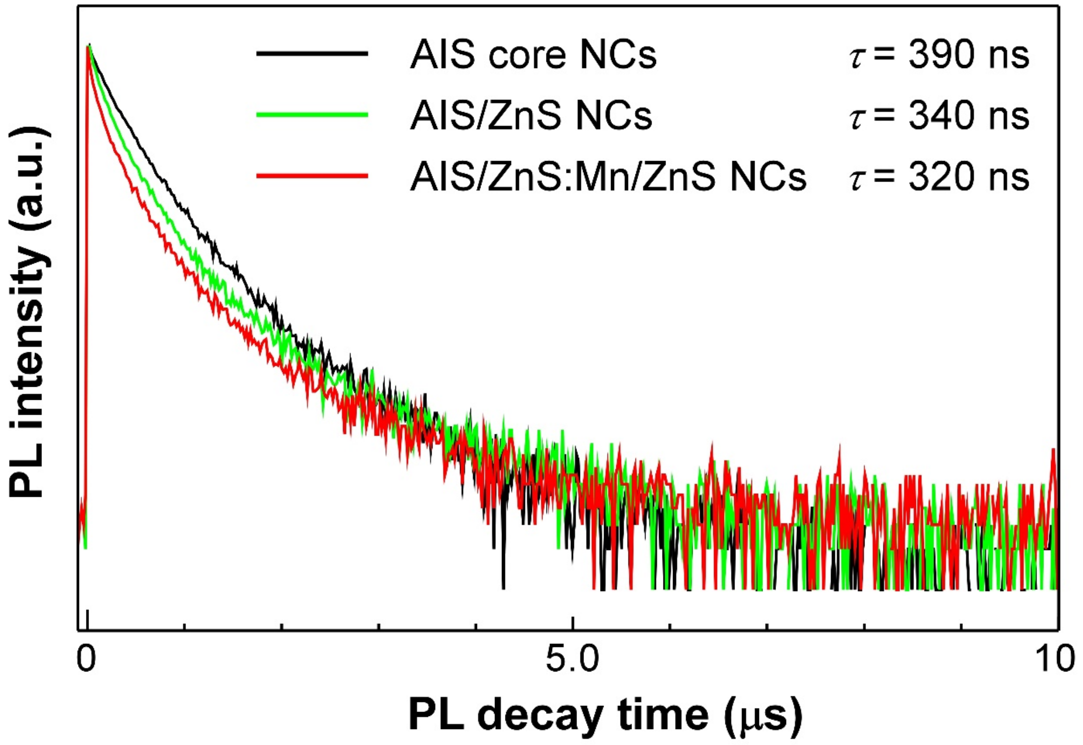

Figure S5. PL decay curves of solution of AIS core NCs, AIS/ZnS NCs, and AIS/ZnS:Mn/ZnS NCs by optical excitation with 365 nm light. The PL decay measurements of AIS core NCs, AIS/ZnS NCs, and AIS/ZnS:Mn/ZnS NCs were detected at 608 nm, 538 nm and 508 nm, respectively.

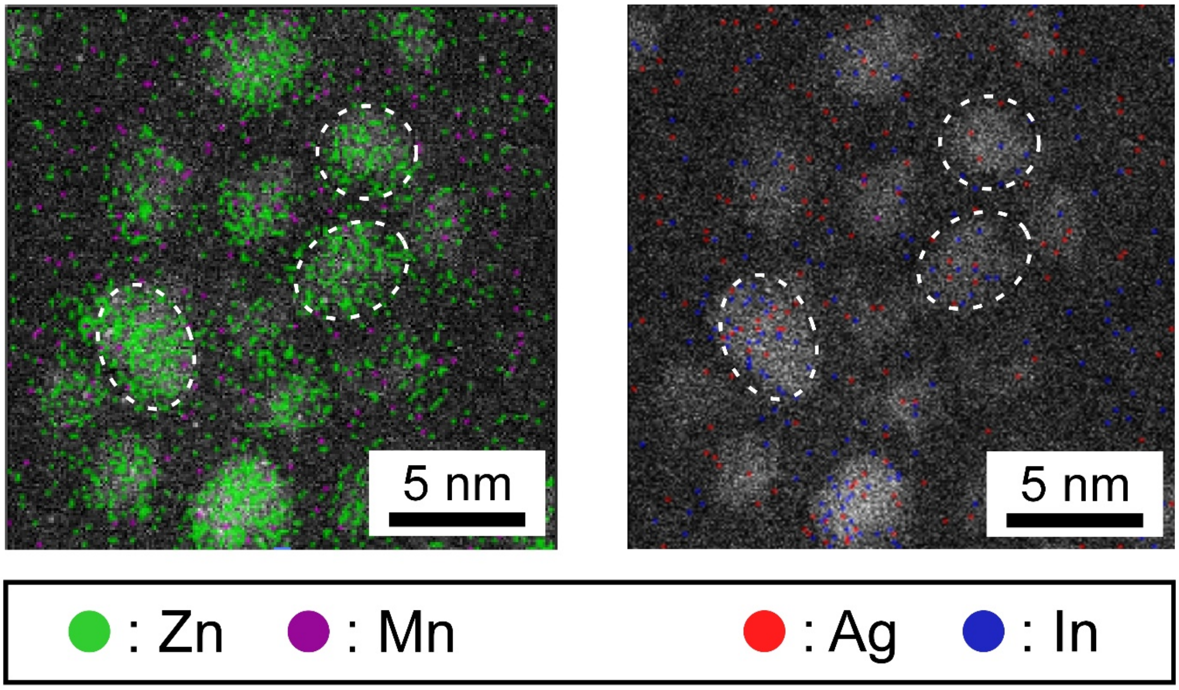

Figure S6. Microscopic elemental mapping images of AIS/ZnS:Mn/ZnS NCs observed by the ARM. Green, purple, red and blue dots in the images indicate Zn, Mn, Ag and In, respectively, and the circular spots correspond to the single NCs.

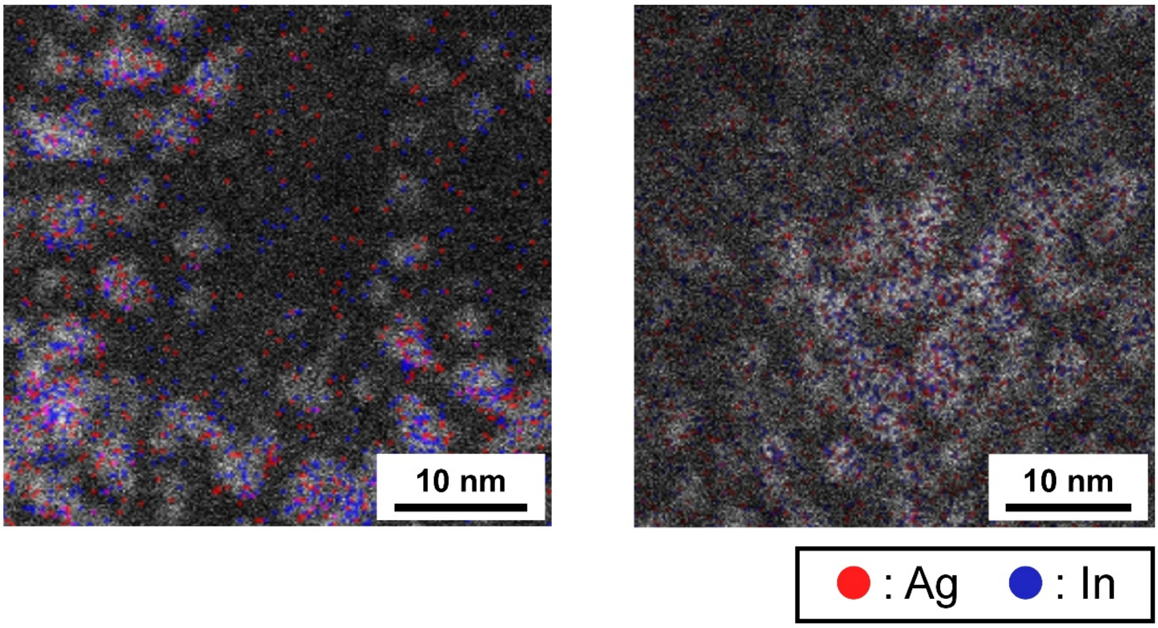

Figure S7. Microscopic elemental mapping images of AIS/ZnS:Mn/ZnS NCs observed by the ARM. Red and blue dots in the images correspond to Ag and In, respectively. The two areas in this figure were different from the Figure 3b and Figure S6.
